# Identification and characterization of novel *Plasmodium falciparum* cyclophilins and their roles in the antimalarial actions of cyclosporin A and derivatives

**DOI:** 10.1186/1475-2875-9-S2-O3

**Published:** 2010-10-20

**Authors:** Alejandro Marin-Menendez, Angus Bell

**Affiliations:** 1 Department of Microbiology, School of Genetics and Microbiology, Moyne Institute of Preventive Medicine, Trinity College Dublin, Dublin 2, Ireland

## Background

**Figure 1:**
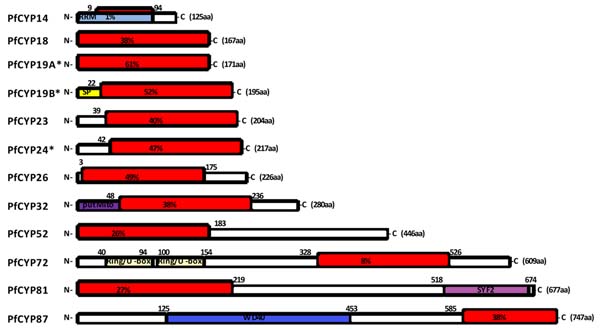
Domain architectures of *P. falciparum* cyclophilin and cyclophilin-like proteins annotated in PlasmoDB. Differently-coloured boxes indicate recognizable domains; red boxes indicate cyclophilin/cyclophilin-like domains with numbers indicating *%* identities to human cyclophilin A(hCYP18). RRM, RNA recognition motif; SP, signal peptide; putMito, putative mitochondrial signal; SYF2, splicing factor 2; WD40, WD40 domain.

Cyclophilins are distributed widely among different organisms and are proposed drug targets for a number of diseases including HIV and hepatitis C infection and ischemia. Cyclophilins play roles in folding and chaperoning of cellular proteins and are the major receptors for the immunosuppressive drug cyclosporin A (CsA). CsA and certain non-immunosuppressive derivatives (e.g., valspodar) possess potent antimalarial activity. We are interested in the role (if any) played by cyclophilins in parasite killing by cyclosporins. We, and others, have previously characterized two CsA-binding cyclophilins (PfCYP19A and PfCYP19B) but a family of genes encoding uncharacterized cyclophilins/cyclophilin-like proteins is also seen in the *P. falciparum* genome (Figure 1).

## Results and conclusions

All but three of the cyclophilin/cyclophilin-like genes (or in the case of the larger proteins, their CYP domains) were cloned identically into a pET vector encoding a C-terminal His_6_-tag and eight of them were successfully expressed in *Escherichia coli* and purified using nickel-chelate affinity chromatography. All of the recombinant proteins showed chaperone activity on model substrates, while only PfCYP19A and PfCYP19B demonstrated peptidyl-prolyl isomerase (foldase) activity and were bound by CsA. Our data suggest the existence of a cyclophilin-type chaperone family whose partner proteins are not yet known but might include proteins exported to the host erythrocyte.

